# A Thrombomodulin Gene Polymorphism (C1418T) Is Associated with Early Outcomes in Patients Undergoing Coronary Artery Bypass Graft Surgery with a Conventional Cardiopulmonary Bypass during Hospitalization

**DOI:** 10.3390/medicines4020022

**Published:** 2017-04-23

**Authors:** Ching-Chou Pai, Yi-Wen Lin, Yi-Ting Tsai, Shih-Hurng Loh, Chih-Yuan Lin, Chin-Sheng Lin, Yi-Chang Lin, Hung-Yen Ke, Feng-Yen Lin, Chien-Sung Tsai

**Affiliations:** 1 Division of Cardiovascular Surgery, Department of Surgery, Show-Chwan Memorial Hospital, Changhua 500, Taiwan; actirin1945@gmail.com; 2 Division of Cardiovascular Surgery, Department of Surgery, Chi-Mei Medical Center, Tainan 710, Taiwan; 3 Division of Cardiovascular Surgery, Tri-Service General Hospital, National Defense Medical Center, Taipei 114 , Taiwan; ywlin7@gmail.com (Y.-W.L.); cvsallen@mail.ndmctsgh.edu.tw (Y.-T.T.); linrock@ms26.hinet.net (C.-Y.L.); m860630@office365.ndmctsgh.edu.tw (Y.-C.L.); drkehy@yahoo.com.tw (H.-Y.K.); 4 Institute of Oral Biology, National Yang-Ming University, Taipei 112, Taiwan; 5 Division of Cardiology and Cardiovascular Research Center, Taipei Medical University Hospital, Taipei 110, Taiwan; 6 Department and Graduate Institute of Pharmacology, National Defense Medical Center, Taipei 114, Taiwan; shloh@ndmctsgh.edu.tw; 7 Division of Cardiology, Tri-Service General Hospital, National Defense Medical Center, Taipei 114, Taiwan; littlelincs@gmail.com; 8 Department of Internal Medicine, School of Medicine, College of Medicine, Taipei Medical University, Taipei 110, Taiwan; 9 Division of Cardiovascular Surgery, Department of Surgery, Taoyuan Armed Forces General Hospital, Taoyuan 325, Taiwan

**Keywords:** throbomodulin, cardiopulmonary bypass, polymorphism

## Abstract

**Background:** Thrombomodulin (TM) is a type of cell membrane-bound anticoagulant protein cofactor in the thrombin-mediated activation of protein C. Previous evidence has shown an association between TM polymorphisms and systemic inflammation. Conventional cardiopulmonary bypass (CPB), beating-heart CPB, and off-pump techniques have been widely used in cardiac surgery. However, these techniques may also cause systemic inflammatory responses in the patients. Whether TM polymorphisms are associated with systemic inflammation after cardiac surgery is still unclear. **Methods:** We analyzed the TM gene C1418T polymorphisms in 347 patients who underwent coronary artery bridge graft (CABG) surgery using allele-specific primers in a PCR assay. The clinical data during the hospital stay were collected and tested for correlations with the TM gene C1418T polymorphisms. **Results:** We separated the patients into two groups based on their TM C1418T genotype (CC genotype group and CT/TT genotype group). The days spent in an intensive care unit (ICU) and the incidence of fever in the ICU were significantly lower in the beating-heart CPB and off-pump groups than in the conventional CPB group. Additionally, the TM gene C1418T polymorphisms did not affect the early outcomes in patients in the beating-heart CPB and off-pump groups. Interestingly, in the conventional CPB group, patients with the CC genotype had a lower rate of fever, shorter duration of fever, and delay of ICU when compared with the CT/TT genotype. **Conclusion:** Surgeons may use a patient’s TM gene C1418T polymorphism to predict the strength of systemic inflammation and speculate on early outcomes during hospitalization before conventional CPB is performed.

## 1. Introduction

Percutaneous coronary intervention (PCI) is widely used to treat cardiovascular diseases. However, recent evidence has demonstrated that coronary artery bridge graft (CABG) surgery plus medical treatment is a more efficacious strategy to rebuild vessel systems, decrease strokes by 50%, and reduce mortality by 20% than PCI plus medical treatment in patients with diabetes mellitus [1]. Therefore, the most important technique; that is, cardiopulmonary bypass (CPB), will continue to be used extensively to support the process of CABG surgery, even though systemic inflammation caused by CPB cannot be avoided [2,3,4,5]. In addition to exploring methods to control postoperative inflammation, cardiac surgeons have developed off-pump techniques and hybrid techniques for on-pump beating-heart CPB (beating-heart CPB) for coronary surgery, in order to reduce systemic inflammatory responses [6,7]. Although the off-pump technique and beating-heart CPB have significantly reduced inflammation, they are still not entirely suitable for many patients who have a critical cardiac situation. In short, how to select the most appropriate technique for patients who will undergo cardiac surgery is a major responsibility for the surgeon.

Thrombomodulin (TM) plays an essential role in maintaining normal endothelial cell function [8]. The TM form present on most normal endothelial cells has high affinity receptors for thrombin [9]. The TM-thrombin complexes might modulate the activation of protein C in association with coagulation [10,11]. Additionally, TM is also expressed on keratinocytes, megakaryocytes, platelets, monocytes [12], neutrophils, and smooth muscle cells [13]. Previous studies have shown that abnormal TM expression is associated with many diseases in humans, especially cardiovascular diseases [14]. Monocytes express TM [12], which is affected in a wide variety of pathophysiological conditions. TM-coupled thrombin can activate protein C and inhibit the cytokines by monocytes [15]. The abnormal expression of TM is associated with disturbances of cell function and inflammation. Recently, we demonstrated that TM regulates monocyte migration with interleukin-6-stimulation [16], and the expression of TM on monocytes is associated with early outcomes in patients who received CABG surgery [17]. In mice, the transgenic expression of human TM has anti-coagulant and anti-inflammatory effects, resulting in the protection of allograft vasculopathy [18]. In clinical situations, plasma-soluble TM levels are elevated in patients who have undergone hemodialysis, while levels are significantly decreased after kidney transplantation. Additionally, gene polymorphisms of TM are a factor in the abnormal expression of TM. The TM gene C1418A polymorphism encodes a Ala455Val substitution in the TM protein, which has been associated with the risk of coronary heart disease [19]. The GG-9/-10AT, G-33A, and C-133A mutations in the promoter region may constitute risks for myocardial infarction [20,21] and drive the downregulation of the TM gene and soluble plasma TM levels, which may result in venous thromboembolic disease and carotid atherosclerosis [22]. Although the expression of TM is a proposed endothelial damage biomarker and is associated with early outcomes of patients undergoing CABG surgery, there is little evidence concerning the relationship between single nucleotide polymorphisms (C1418T) in the TM gene and early outcomes of patients undergoing cardiac surgery. Therefore, in this study, we analyzed the TM gene polymorphism C1418T using an allele-specific PCR assay in 347 patients who underwent CABG surgery. We also collected clinical data concerning early outcomes in hospital days and tested for any correlations between patient outcomes and C1418T polymorphisms of the TM gene.

## 2. Materials and Methods

### 2.1. Ethics and Patients

The ethics committee of our institution approved this study (TSGHIRB No. 099-05-150). Written informed consent was obtained from 347 patients undergoing elective CABG surgery from May 2008 to May 2011. The patients were randomly assigned to conventional CPB, beating heart CPB, and off-pump technique groups. Anesthesia and heparinization before the operation and radial artery catheterization was performed, and a Swan-Ganz standard thermodilution pulmonary artery catheter (Abbott Laboratories, Abbott Park, Ill) was inserted through the internal jugular vein. Anesthesia for all patients was induced with thiopental and maintained with isoflurane in oxygen, fentanyl, and pancuronium. Porcine heparin was administered as an anticoagulant before cannulation or before the application of the stabilization device (the activated coagulation time was more than 480 s with a heparin dose of 300 U/kg). On the discontinuation of the CPB circuit (for the conventional CPB and beating-heart CPB groups) or the completion of proximal anatomies (for the off-pump technique group), heparin was neutralized with protamine sulfate. No antifibrinolytic drugs, such as aprotinin, were used in these patients.

### 2.2. Conventional CPB, Beating-Heart CPB Technique and Off-Pump Technique

All patients underwent median sternotomy CABG. The CPB was performed with standard cannulation and a Capiox SX 18 extracorporeal membrane oxygenator (Terumo, Irvine, CA, USA). A nonpulsatile Terumo Sarns 9000 roller pump (Terumo, Irvine, CA, USA) was used for all patients. The invasive blood pressure was monitored with an arterial catheter during the surgical procedure. The MAP of all patients placed on CPB was maintained at approximately 70 mmHg with pharmacological treatment and/or a heart-lung machine/mechanical support. For the conventional CPB group, patients were cooled to 28 °C to 30 °C body temperature, and cardiac arrest was induced with warm blood cardioplegia (the ratio of cardioplegia solution to blood was 1:4; 15 mL/kg body weight), which was delivered antegradely and retrogradely every 20 min. Patients were maintained at 35 °C to 37 °C in the beating-heart CPB group. The saphenous vein and intermittent anastomosis of the graft were used for revascularization.

Patients underwent beating-heart CPB using a stabilization device (Guidant, Santa Clara, CA, USA) for the immobilization of the myocardial surface. Aortic-side anastomoses were performed after completing the distal anatomies with the assistance of a partial occlusion aortic clamp. For the off-pump technique group, a Guidant stabilization device was used on the myocardial surface for the distal anastomoses.

After completion of the distal anastomoses, the proximal anastomoses were performed with the assistance of a partial occlusion aortic clamp. Patients were maintained at 35 °C to 37 °C in this group.

### 2.3. Biolaboratory Studies of Patients Undergoing Elective CABG Surgery

Patients were excluded from the study if they had undergone previous isolated cardiac surgery or experienced a reduced cardiac ejection fraction (less than 50%), if they had a history of cardiogenic shock, or if they had used an intra-aortic balloon pump (IABP), received extracorporeal membrane oxygenation, or had been placed on a respiratory ventilator. Patients with rheumatoid arthritis, asthma, chronic bronchitis, cancer, or autoimmune disease, or patients receiving steroidal or nonsteroidal anti-inflammatory drug therapies, were also excluded. Aspirin was discontinued in all patients seven days before the operation. Blood was obtained from an indwelling arterial catheter at regular time points. All blood samples were collected into tubes containing 3.8% sodium citrate. The blood samples were collected pre-incision and 24 h after the commencement of the operation. The white blood cell and platelet contents were measured.

### 2.4. Extraction of Genomic DNA

Patients’ peripheral leukocytes were isolated from the blood samples before the operation processes were performed. Genomic DNA was purified using the EasyPure genomic DNA purification kit (Bioman Inc. Taipei, Taiwan). Cut-off pipette tips and wide-bore pipettes were used during the process to minimize DNA breakage. Precipitated DNA was dissolved in 1X TE (10 mM Tris, 1 mM EDTA, pH 7.9), and the purity and concentration of the DNA were determined using a Nanodrop 2000 spectrophotometer (Thermo Scientific, Wilmington, DE, USA). High quality DNA was reflected by a consistent 260 nm/280 nm absorbance ratio of 1.8–2.0. The genomic DNA solution was stored at 4 °C until use.

### 2.5. Allele-Specific Primer PCR (ASP-PCR) Assay

We developed allele-specific primer PCR (ASP-PCR) for the detection of the C1418T (Ala455Val) polymorphism in the TM gene. A common forward primer, 5′-TCAGAGCCAACTGCGAGTACC-3′, and two allele-specific reverse primers, C Allele 5′-ACAGTCGGTGCCAATGTGGCGTG-3′ and T Allele 5′-ACAGTCGGTGCCAATGTGGCGTA-3′, were used. Reactions were performed in a Biometra T Gradient thermocycler (Biometra, Goettingen, Germany) with an initial heating at 95 °C for 5 min, followed by 30 cycles of 95 °C for 20 s, 66 °C for 20 s, and 72 °C for 30 s, and a final incubation at 72 °C for 5 min. A 332 bp amplification product was detected. A control forward primer, 5′-TTGCAACCAG ACTGCCTGTCC-3′, and a control reverse primer, 5′-CGCACGTGCTGCAGCACT AC-3′, were used to amplify a 720 bp fragment containing the polymorphic site to serve as an internal positive amplification control. The products were separated by electrophoresis on 1% agarose gels and photographed under UV light. DNA containing the C genotype only generated a PCR product when the C allele-specific reverse primer was included in the PCR reaction; the T genotype’s DNA only produced a PCR fragment when the T allele-specific reverse primer was added into the PCR reaction.

### 2.6. Restriction Fragment Length Polymorphism (RFLP) Analysis

To further confirm the genotyping results obtained from ASP-PCR, RFLP analysis specific for the TM C1418T polymorphism was developed. Briefly, PCR was performed with a forward primer, 5′-CAACCAGACTGCCTGTCCAGC-3′, and a mutated reverse primer, 5′-CAGTCGGTGCCAAT GTGTCGG-3′ (the underlined nucleotide introducing a C to A modification in the coding strand), to introduce a restriction site for the Hyp188I enzyme. Reactions were performed with an initial heating at 95 °C for 5 min, followed by 35 cycles of 95 °C for 20 s, 58 °C for 20 s, and 72 °C for 30 s, and a final incubation at 72 °C for 5 min, generating an amplification product of 214 bp. After confirmation of a successful PCR process, 3 µL of the PCR product was digested with the Hyp188I enzyme at 37 °C for 4 h. The digested products were separated by electrophoresis on 3% agarose gels. Finally, visualization of the RFLP was performed under UV light. A Hyp188I site is only present in the PCR product generated by the T allele and is not in that produced by the C allele. Therefore, we could easily differentiate between the two polymorphisms according to the digestion patterns. The PCR product produced by the T allele was cut into 196 bp and 18 bp fragments after Hyp188I digestion, but the 214 bp PCR product of the C allele remained intact.

### 2.7. Statistical Analyses

We separated the patients into six groups: Group 1, patients who underwent conventional CPB and with the CC genotype of TM; Group 2, patients who underwent conventional CPB with the CT or TT genotype of TM; Group 3, patients who underwent the beating-heart CPB technique with the CC genotype of TM; Group 4, patients who underwent a beating-heart CPB technique with the CT or TT genotype of TM; Group 5, patients who underwent the off-pump technique with the CC genotype of TM; and Group 6, patients who underwent the off-pump technique with the CT or TT genotype of TM. All values are expressed as the mean ± SEM. Statistical evaluation was performed using a Student’s *t*-test and a one-or two-way ANOVA followed by a Dunnett’s test. For all statistical evaluations, differences in the data with *p*-values < 0.05 were considered statistically significant.

## 3. Results

### 3.1. TM C1418T Polymorphism in Patients Who Underwent Elective CABG

In this study, we obtained the TM C1418T polymorphism from ASP-PCR and RFLP analysis using a specific primer. The numbers of patients with the CC, CT, or TT polymorphism are shown in Table 1. In the conventional CPB group, there were 116 patients with the CC genotype, 62 patients with the CT genotype, and 20 patients with the TT genotype. In the beating-heart CPB group, 41 patients with the CC genotype, 19 patients with the CT genotype, and nine patients with the TT genotype. In the off-pump technique group, there were 44 patients with the CC genotype, 28 patients with the CT genotype, and eight patients with the TT genotype. To perform further statistical analysis, we combined the patients carrying the CT and TT genotypes into a single group in the respective surgery group.

### 3.2. Patients with Similar Characteristics before Elective CABG Were Assessed

We assessed the relative influences of conventional CPB, beating-heart CPB, and the off-pump technique on the clinical outcomes and laboratory biomarkers. Table 2 shows the demographic data. Patients undergoing CABG with conventional CPB were not significantly different from those undergoing CABG with beating-heart CPB or the off-pump technique in terms of age, body weight, body height, or preoperative left ventricle ejection fraction. None of the patients had experienced a previous stroke or myocardial infarction, and none required pharmacological intervention, including inotropic agents or mechanical support, including IABP or extracorporeal membrane oxygenation before the operation. The rates of hypertension, hypercholesterolemia, diabetes mellitus, and peripheral vascular diseases did not differ significantly across all groups.

### 3.3. Patients Encountered Perioperative Characteristics in Elective CABG

As shown in Table 3, among the patients treated with CPB, the total CPB times were 130.5 ± 48.7, 126.8 ± 50.1, 119.3 ± 49.6, and 123.0 ± 30.5 min for the CC genotype, CT/TT genotype in the conventional CPB group, and CC and CT/TT genotypes in the beating-heart CPB groups, respectively. In the conventional CPB group, the aortic clamping time was 65.8 ± 20.5 and 71.5 ± 13.9 min, and the esophageal temperature was maintained at 27.5 ± 2.5 and 27.5 ± 1.9 °C for the CC genotype and CT/TT genotype patients, respectively. In the beating-heart CPB and off-pump technique groups, the patient’s esophageal temperature was maintained as shown in Table 3, respectively. The mean number of units of heparin received was lower in the off-pump technique group than in the conventional CPB (21,500 ± 2286 units) or beating-heart CPB groups (20,500 ± 3900 units). There were no significant differences in the numbers of grafts used in the three groups.

### 3.4. Blood and Biochemical Data in Elective CABG Patients

Table 4 showed the blood and biochemical analysis data. In our study population, the percentages of circulating neutrophils, basophils, eosinophils, monocytes, and platelets were not significantly different between the six groups at the time point of pre-surgery and post-surgery. In contrast, the rations of lymphocytes were decreased after surgery in the CPB groups with the CC or CT/TT genotype (conventional CPB group and beating-heart CPB group).

Biochemical analyses showed that the plasma concentrations of the MB isoform of creatine kinase (CK-MB) and the mean CK-MB value did not deteriorate after surgery in all groups. However, troponin I levels were significantly elevated in the conventional CPB group after surgery (in the CC patients: pre-surgery: 3.5 ± 2.0 mg/L and post-surgery: 15.4 ± 4.6 mg/L, *p* = 0.018; in the CC/CT patients: pre-surgery: 4.2 ± 2.0 mg/L and post-surgery: 20.4 ± 6.1 mg/L, *p* = 0.013). In the beating-heart CPB and off-pump technique groups, the levels of troponin I had an increasing trend post-surgery, but did not reach statistical significance. Additionally, the elevation of troponin I was not associated with the TM C1418T polymorphism in a group of patients who underwent the same surgical procedure.

Kidney function (blood urea nitrogen [BUN] and creatinine) and liver function (aspartate aminotransferase [AST] and alanine transaminase [ALT]) did not deteriorate after surgery in all groups. It is well known that serum TM and C-reactive protein (CRP) are analyzed to monitor systemic inflammation after cardiac surgery; therefore, the level of serum TM and CRP were also determined. Except for CC/CT in the conventional CPB group with increasing serum CRP levels (at pre-surgery: 2.6 ± 1.2 mg/L and at post-surgery: 8.4 ± 2.1 mg/L, *p* = 0.018), both CC in the conventional CPB group (pre-surgery: 3.0 ± 1.0 mg/L and post-surgery: 9.0 ± 3.2 mg/L, *p* = 0.075) and CC and CT/TT in the beating-heart CPB group and CC and CT/TT in the off-pump technique groups, did not have higher CRP values post-surgery compared to those pre-surgery. Patients in the CC and CT/CT groups who underwent conventional CPB or the beating-heart CPB technique had higher TM levels post-surgery compared to those pre-surgery (CC patients in the conventional CPB group: pre-surgery: 56.3 ± 18.4 ng/mL and post-surgery: 123.5 ± 2.5 ng/mL, *p* = 0.032; CC/CT patients in the conventional CPB group: pre-surgery: 62.1 ± 20.1 ng/mL and post-surgery: 130.4 ± 21.0 ng/mL, *p* = 0.020; CC patients in the beating-heart CPB group: pre-surgery: 49.7 ± 25.4 ng/mL and post-surgery: 110.3 ± 16.4 ng/mL, *p* = 0.048; CC/CT patients in the beating-heart CPB group: pre-surgery: 55.3 ± 19.7 mg/L and post-surgery: 123.4 ± 19.7 ng/mL, *p* = 0.018). The levels of serum CRP and TM were not associated with the TM C1418T polymorphism in a group of patients who underwent the same surgical procedure after surgery. These results indicated that surgical procedures influence the expression of CRP and TM after cardiac surgery, but not the TM C1418T polymorphism in patients.

### 3.5. Patients with the TM CC Genotype Provide Better Early Outcomes during Hospitalization Than Those with the CT/TT Genotype in the Conventional CPB Group

As shown in Table 5, the mean number of packed red blood cells (pRBCs) received showed a decreasing trend in the off-pump technique group (CC genotype group: 2.2 ± 1.2 and CT/TT genotype group: 2.5 ± 1.0 units, respectively) when compared to the conventional CPB (CC genotype group: 5.0 ± 2.7 and CT/TT genotype group: 5.2 ± 3.1 units, respectively) or beating-heart CPB groups (CC genotype group: 4.0 ± 2.6 and CT/TT genotype group: 4.1 ± 2.2 units, respectively), although statistical significance was not found. Patients in the conventional CPB group exhibited a higher drainage loss (CC genotype group was 986.1 ± 123.7 and CT/TT genotype group was 980.1 ± 104.7 mL, respectively) than that in the beating-heart CPB (CC genotype group: 382.6 ± 101.6 and CT/TT genotype group: 399.6 ± 121.8 mL, respectively) and off-pimp technique groups (CC genotype group was 330.7 ± 121.3 and CT/TT genotype group was 288.7 ± 91.9 mL, respectively). Additionally, there were no significant differences in the numbers of pRBCs used and the drainage loss between the CC genotype and CT/TT genotype groups in the same surgical procedure group. The mean number of days spent in the intensive care unit (ICU) and the incidence and duration of any fever (ear temperature more than 37.5 °C) was significantly lower in the beating-heart CPB and off-pump technique groups than in the conventional CPB group. Interestingly, in the conventional CPB group, patients with the CC genotype had a lower rate of fever, shorter durations of fever, and a delay of ICU when compared with the CT/TT genotype.

No patients died while in the hospital. This result suggests that patients with the TM CT or TT polymorphism may face higher inflammation while undergoing conventional CPB cardiac surgery

## 4. Discussion

In this study, although there were elevated levels of TM in the plasma after surgery, both in the conventional CPB and beating-heart CPB groups, only patients in the conventional CPB group had clear harms (a higher incidence of ICU fever and longer ICU stay). Our statistical analyses demonstrated that the C1418T polymorphism of the TM gene may be a potential factor in the duration of ICU stays and the rates of ICU fever in the conventional CPB group patients as early outcomes. In contrast, no matter what the TM genotype (CC, CT, or TT) was, there was no significant difference in the early outcomes in the beating-heart CPB and off-pump technique groups.

TM plays critical roles in regulating inflammatory responses. Additionally, TM might prevent the disturbance of endothelial cells by suppressing the expression of the vascular cell adhesion molecule-1 (VCAM-1) and preventing the activation of leukocytes. The TM protein contains an *N*-terminal globular domain, which has been shown to interfere with complement activation and confer protection from neutrophil-mediated tissue damage [23]. TM may protect the cells by increasing complement factor I-mediated inactivation of C3b, binding thrombin, and preventing C5 activation [24]. Monocyte activation plays a potential role during CABG with conventional CPB [25,26]. CPB changes the inflammatory capacity of monocytes [4]. TM is expressed constitutively on the surfaces of resting monocytes and macrophages [27]. Recently, we showed that domain 5 of TM co-localized with the cytoskeleton, F-actin, and intersectin I when the monocytes were stimulated by IL-6 [16], which is associated with cell migration and inflammatory responses [28,29,30]. TM concomitantly exhibits procoagulant activity and adhesion molecules expressing microparticles [31], which may be a key regulator of monocyte-related coagulation reactions. TM expression is also altered by several pathophysiological situations and biological factors on monocytes [32,33]. The downregulation of TM may yield inflammation [32], as well as coagulopathy, via the decrease of activated protein C after cardiac surgery [34,35]. Additionally, the transgenic expression of human TM has anti-coagulant and anti-inflammatory effects, resulting in the protection of allografts [18]; plasma-soluble TM levels are elevated in patients who have undergone hemodialysis, while levels are significantly decreased after kidney transplantation. Recently, although we have previously demonstrated that the blood levels of TM are not associated with the occurrence of vasculopathy, a correlation between the TM C1418T polymorphism and the development of vasculopathy after heart transplantation was observed [36].

Previous studies have shown that abnormal TM expression is associated with several cardiovascular diseases in humans [14]. Polymorphisms and mutations of the TM gene are considered to be associated with the abnormal expression of the TM protein. Since the mutation in the TM gene was identified, several other point mutations in the TM gene have been identified, including G127A, G236C, G543A, G1456T, and C1502T, in patients associated with venous thrombosis and cardiovascular diseases [37]. The TM gene C1418A (Ala455Val) polymorphism predicts the risk of developing coronary heart disease [19] in African American patients. In contrast, five mutations (GG-9/-10AT, G-33A, and C-133A) that were identified in the promoter region of the TM gene may constitute risks for myocardial infarction [20] in the Chinese population. The G-33A and C-133A mutations downregulate the expression of the TM gene and lower the concentration of the plasma TM protein, which results in carotid atherosclerosis, venous thromboembolic disease, and the onset of acute myocardial infarction [21,22] in smokers. Additionally, the occurrence and influence of the TM polymorphism may be race-dependent [19]. Individuals, especially young patients in Taiwan, with the G-33A polymorphism carry a higher risk of acute myocardial infarction [38]. Similarly, in Koreans, the G-33A polymorphism of the TM gene may be a genetic risk factor for myocardial infarction as a result of abnormal promoter activity [39]. In fact, we also analyzed the influences of the G-33A polymorphism of the TM gene in patients with coronary artery bypass graft surgery, and acquired a negative result in this study. In the Japanese population, those with A2729C and C1418T of the TM gene have a higher risk of developing deep vein thrombosis [40]. Furthermore, the relationship between the C1418T polymorphism and the occurrence of cardiovascular disease is diverse. Ohlin et al. showed that people with the C/T dimorphism were at a higher morbidity and mortality risk of coronary heart disease and myocardial infarction [41]. In contrast, Aleksic et al. reported that the C1418T polymorphism of the TM gene did not decrease the plasma level of TM and was not associated with cardiovascular diseases [42]. In this study, after surgery, the expression of CRP was higher compared to that before surgery in patients with the CT/TT genotype in the conventional CPB group. However, patients with the CC or CT/TT genotype in the beating-heart CPB and off-pump technique groups, and the CC genotype in the conventional CPB groups without significant CRP changes after cardiac surgery, were processed. Although the correlation between the CT/TT genotype of TM and increased CRP levels is unknown, we do know that patients with the CT or TT genotype of TM may suffer from higher levels of systemic inflammation during CABG surgery with conventional CPB. Whether the TM polymorphism influences the production of CRP in hepatocytes, which mediates systemic inflammation after surgery, needs to be elucidated. The expression of TM showed more specificity on monocytes than granulocytes and lymphocytes [12,17,33], and we also found that the C1418T polymorphism of the TM gene regulates the early outcomes of patients in the conventional CPB group in this study. Therefore, we speculate that the alteration of the 455th amino acid from alanine to valine in the TM protein sequence resulting from C1418T polymorphism changes may influence the interaction of TM with the cytoskeleton when conventional CPB is performed. Additionally, patients with the CT/TT genotype of the TM gene in the conventional CPB group experience a higher incidence of ICU fever and longer ICU stays than those with the CT/TT genotype of the TM gene in the beating-heart CPB group, and we are currently conducting studies to investigate this possibility.

CPB increases cytokine production and postoperative inflammation may result from surgical trauma, the attachment of foreign surfaces by the oxygenator/CPB tube, and ischemia–reperfusion injury. However, a previous report compared the inflammatory response in cardiac revascularization between the conventional CPB, beating-heart CPB, and off-pump techniques [43]. Gulielmos et al. also explored IL-6 expression in a prospective randomized comparison of different surgical techniques. They considered surgical trauma to be a strong trigger of the inflammatory response [44]. However, in this study, we also aimed to distinguish the influences of foreign surface attachment by the oxygenator/CPB tube and cardiac arrest/ischemia-reperfusion injury by comparing the CRP and serum TM levels, as well as the clinical parameters of early outcomes during conventional CPB, beating-heart CPB, and the off-pump technique in patients undergoing CABG. The concentration of plasma TM was always increased in the conventional CPB and beating-heart CPB groups, but not in the off-pump technique group after surgery, indicating that the attachment of the rolling pump/foreign surface increased TM production and TM fractured off from the cell membrane, even though we do not know the original main source of the TM. Additionally, in the conventional CPB group, but not in the beating-heart CPB and off-pump technique groups, CRP markedly increased after surgery, indicating that cardiac arrest/ischemia–reperfusion injury plays a critical role in the changes of the serum CRP levels. Interestingly, although there were no significant differences in the CRP and TM levels after surgery between the patients with CC, CT, and CT genotypes of the TM gene in the conventional CPB group, we are the first group to demonstrate that the role of the C1418T polymorphism in the TM gene is critical in postoperative inflammation with conventional CPB. In further studies, an analysis of the relationship between the C1418T polymorphism of the TM gene and immunity may lead to a more accurate understanding of postoperative inflammation in conventional CPB.

In this study, patients with the CT/TT genotype of the TM gene had a higher rate of fever, longer duration of fevers, and a delay of ICU when compared with the CC genotype of the TM gene in the conventional CPB group. Additionally, the expression of CRP increased after surgery in patients with the CT/TT genotype in the conventional CPB group, but not in patients with the CC or CT/TT genotype in the beating-heart CPB and off-pump technique groups or the CC genotype in the conventional CPB groups. To date, we are not aware of the mechanisms underlying this phenomenon; we only know that patients with the CT or TT genotype of TM may suffer from higher levels of systemic inflammation during CABG surgery with conventional CPB. Fortunately, in the clinic, although patients with the CT/TT genotype of the TM gene underwent a severe recovery process after cardiac surgery with conventional CPB, there were no proven significant acute neurological disturbances, cognitive impairments, or complicated complications. On the other hand, even though we have not acquired enough evidence regarding this phenomenon, these pioneer/primary explorations may encourage the study of a larger cohort to improve our understanding of the actual mechanism of post-CPB inflammation in the future, without neglecting the judicious judgment of the patient's pre-existing endogenous situation in determining the most appropriate surgical technique for their own optimal outcome. Additionally, if the surgeon can anticipate the recovery of patients after cardiac surgery and control it, it will not only increase the quality of treatment during hospitalization, but will also reduce the cost of medical expenses and insurance payments, as well as reduce the financial burden on the patient’s family and the government. This is an important issue for developed countries around the world.

In conclusion, we compared the inflammatory impact of the C1418T polymorphism of the TM gene in CABG surgery with the conventional CPB, beating-heart CPB, and the off-pump techniques. We believe that better early outcomes for patients undergoing conventional CPB might be associated with the CC genotype of the TM gene. This expression could prove to be a predictor of the degree of inflammation during CABG surgery with the conventional CPB procedure. In addition, it may be a promising therapeutic approach for treating patients with inflammation after CABG surgery.

## 5. Limitations

We included 347 patients and randomly assigned them to three groups (conventional CPB, beating-heart CPB, and off-pump technique) for CABG surgery. All surgeries were performed by two surgeons. Since all data came from a single medical center with two attending physicians, there was the possibility of bias. Additionally, it was very difficult to carry out a clinical study with a sufficient number of cases at a single medical center in Taiwan. Therefore, we combined the patients carrying the CT and TT genotypes into a single group and examined the differences between this group and the CC genotype group. Although we achieved positive results with reference to the TM C1418T polymorphism being associated with early outcomes in patients who underwent conventional CPB-CABG surgery, we still intend to identify more patients and follow up on their long-term recovery in future studies. This approach should increase the reliability of our statistics and may provide better predictions of early outcomes before the surgeries are performed.

## Figures and Tables

**Table 1:** Patients (total 347) with CC, CT, or TT genotype.

Operation	CABG Surgery with Conventional CPB	CABG Surgery with Beating-Heart CPB	CABG Surgery with Off-Pump Technique
**CC** **Genotype**	e116	41	44
**CT** **Genotype**	62	19	28
**TT Genotype**	20	9	8
**Total**	198	69	80

**Table 2:** Demographic characteristics (*n* = 347) before surgery in elective CABG patients.

Operation	CABG Surgery with Conventional CPB (*n* = 198)			CABG Surgery with Beating-Heart CPB (*n* = 69)			CABG Surgery with Off-Pump Technique (*n* = 80)		
Genotype	CC (*n* = 116)	CT (*n* = 62)	TT (*n* = 20)	CC (*n* = 41)	CT (*n* = 19)	TT (*n* = 9)	CC (*n* = 44)	CT (*n* = 28)	TT (*n* = 8)
Gender (Male/Female)	62/54	32/30	15/5	20/21	13/6	5/4	22/22	18/10	5/3
Age (years)	63.5 ± 9.8	67.8 ± 12.4	62.4 ± 11.5	68.98 ± 14.7	63.5 ± 15.4	70.4 ± 12.4	65.7 ± 11.9	67.1 ± 14.5	68.1 ± 10.9
Body weight (kg)	69.2 ± 11.7	59.7 ± 15.4	67.4 ± 20.1	60.7 ± 16.4	69.9 ± 12.8	59.3 ± 15.3	62.7 ± 9.6	64.1 ± 12.9	55.7 ± 12.8
Body height (cm)	165.8 ± 19.7	158.6 ± 10.5	159.8 ± 19.7	165.1 ± 18.2	160.9 ± 19.7	160.9 ± 16.4	158.3 ± 16.1	160.7 ± 12.7	159.8 ± 11.9
Hypertension (*n*, %)	103, 88.8%	50, 80.64%	17, 85.0%	30, 73.2%	15, 78.9%	7, 77.8%	36, 81.8%	22, 78.6%	6, 75%
Smoke (*n*, %)	31, 26.7%	15, 24.2%	5, 25.0%	12, 29.3%	5, 26.3%	3, 33.3%	14, 31.8%	9, 32.1%	3, 37.5%
Hypercholesterolemia (*n*, %)	65, 56.0%	34, 54.8%	11, 55.0%	18, 43.9%	8, 42.1%	3, 33.3%	26, 59.1%	15, 53.6%	4, 50.0%
Diabetes mellitus (*n*, %)	57, 49.1%	31, 50.0%	11, 55%	18, 43.9%	8, 42.1%	4, 44.4%	20, 45.5%	13, 46.4%	4, 50.0%
Peripheral vascular disease (*n*, %)	13, 11.2%	16, 25.8%	3, 15.0%	8, 19.5%	4, 21.1%	2, 22.2%	11, 25.0%	6, 21.4%	1, 12.5%
COPD (*n*, %)	15, 12.9%	9, 14.5%	5, 25.0%	6, 15.9%	3, 15.8%	2, 22.2%	6, 13.6%	4, 14.3%	2, 25.0%
Old stroke (*n*, %)	0, 0%	0, 0%	0, 0%	0, 0%	0, 0%	0, 0%	0, 0%	0, 0%	0, 0%
Prior myocardial infarction (*n*, %)	0, 0%	0, 0%	0, 0%	0, 0%	0, 0%	0, 0%	0, 0%	0, 0%	0, 0%
E*jection fraction (%)*	62.5 ± 8.2%	61.5 ± 9.7%	58.7 ± 8.9%	61.8 ± 9.2%	64.8 ± 11.7%	62.7 ± 10.5%	58.7 ± 9.1%	58.9 ± 10.4%	59.4 ± 9.0%
IABP (*n*, %)	0, 0%	0, 0%	0, 0%	0, 0%	0, 0%	0, 0%	0, 0%	0, 0%	0, 0%
ECMO (*n*, %)	0, 0%	0, 0%	0, 0%	0, 0%	0, 0%	0, 0%	0, 0%	0, 0%	0, 0%

CABG, coronary artery bypass grafting; CPB, cardiopulmonary bypass; COPD, chronic obstructive pulmonary disease.; IABP, intra-aortic balloon pump; ECMO, extracorporeal membrane oxygenation. Values are mean ± SD.

**Table 3:** Perioperative characteristics (*n* = 347) in elective CABG patients.

Operation	CABG Surgery with Conventional CPB		CABG Surgery with Beating-Heart CPB		CABG Surgery with Off-Pump Technique	
Genotype	CC	CT/TT	CC	CT/TT	CC	CC/CT
Minimal esophageal temperature	27.5 ± 2.5 °C	27.5 ± 1.9	32.2 ± 1.9 ℃	31.5 ± 2.0	34.9 ± 1.6 °C	34.1 ± 1.7
Heparin (Unit)	20,900 ± 2230	21,000 ± 2190	20,500 ± 5657	20,670 ± 5340	19,500 ± 5500	19,800 ± 4500
CPB time (minutes)	130.5 ± 48.7	126.8 ± 50.1	119.3 ± 49.6	123.0 ± 30.5	−	−
Aortic clamping time (minutes)	65.8 ± 20.5	71.5 ± 13.9	−	−	−	−
Number of grafts	3.5	3.7	3.6	3.5	3.6	3.6

CABG, coronary artery bypass grafting; CPB, cardiopulmonary bypass; Values are mean ± SD.

**Table 4:** Blood and biochemical analysis in elective CABG patients.

Operation/ Genotype	CABG Surgery with Conventional CPB				CABG Surgery with Beating-Heart CPB				CABG Surgery with Off-Pump Technique			
	Pre-Surgery		Post-Surgery		Pre-Surgery		Post-Surgery		Pre-Surgery		Post-Surgery	
	CC	CT/TT	CC	CT/TT	CC	CT/TT	CC	CT/TT	CC	CT/TT	CC	CT/TT
**Leukocytes Differenciation**												
Neutrophil (%)	60.3 ± 9.5	65.3 ± 9.4	70.5 ± 6.1	72.1 ± 6.5	65.4 ± 5.7	66.4 ± 6.4	63.5 ± 6.8	68.5 ± 6.7	68.5 ± 6.9	65.3 ± 6.9	66.8 ± 9.7	69.8 ± 9.7
Eosinophil (%)	3.7 ± 1.1	2.6 ± 1.6	3.7 ± 0.9	3.1 ± 0.6	3.6 ± 2.1	3.6 ± 1.4	3.7 ± 2.0	3.8 ± 1.0	3.7 ± 1.3	3.5 ± 1.2	4.0 ± 1.0	3.8 ± 1.0
Basophil (%)	0.7 ± 0.4	0.5 ± 0.2	0.6 ± 0.2	0.6 ± 0.1	0.5 ± 0.2	0.7 ± 0.4	0.5 ± 0.3	0.7 ± 0.1	0.6 ± 0.2	0.7 ± 0.3	0.6 ± 0.1	0.6 ± 0.2
Monocyte (%)	7.5 ± 1.8	6.5 ± 2.1	6.4 ± 2.8	7.6 ± 2.1	6.6 ± 2.4	6.8 ± 1.5	6.1 ± 2.1	7.0 ± 1.8	6.5 ± 2.0	8.1 ± 3.4	7.1 ± 2.0	6.8 ± 1.9
Lymphocyte (%)	29.3 ± 7.2	29.3 ± 7.2	10.3 ± 5.3 *	11.3 ± 5.3 *	30.2 ± 9.5	23.5 ± 9.7	9.8 ± 2.7 *	10.4 ± 6.7 *	23.5 ± 6.7	20.4 ± 6.7	22.5 ± 6	19.8 ± 6.7
Platelet (× 10^10^/L)	22.5 ± 5.4	26.5 ± 5.4	22.3 ± 5.1	20.3 ± 7.9	23.5 ± 9.7	19.8 ± 5.9	22.4 ± 10.5	20.6 ± 9.7	22.7 ± 9.7	20.8 ± 6.7	21.6 ± 9.7	20.4 ± 8.9
**Myocardio Damage**												
CK-MB/CK (%)	5.0 ± 1.3	4.2 ± 1.9	4.9 ± 2.7	5.1 ± 2.4	4.0 ± 1.6	5.0 ± 2.1	4.9 ± 2.0	4.6 ± 1.0	4.6 ± 0.9	4.7 ± 1.1	4.9 ± 1.3	4.7 ± 0.9
CK-MB (mg/dL)	25.4 ± 10.4	27.4 ± 9.4	25.6 ± 10.3	27.9 ± 9.8	20.4 ± 11.3	26.8 ± 10.4	23.5 ± 9.7	26.4 ± 9.8	20.5 ± 6.7	22.5 ± 6.7	25.4 ± 9.8	26.4 ± 6.9
Troponin I (mg/L)	3.5 ± 2.0	4.2 ± 2.0	15.4 ± 4.6 *	20.4 ± 6.1*	5.4 ± 4.5	3.9 ± 3.0	10.5 ± 10.1	11.5 ± 9.1	6.7 ± 5.1	5.4 ± 4.1	12.4 ± 10.0	9.8 ± 5.4
**Kidney Function**												
BUN	20.3 ± 4.5	16.4 ± 3.7	16.9 ± 7.3	20.4 ± 10.3	20.5 ± 6.9	19.6 ± 6.7	23.5 ± 13.7	18.6 ± 5.8	20.5 ± 6.6	21.3 ± 6.9	20.6 ± 10.5	23.5 ± 4.1
Creatinine (mg/dL)	1.0 ± 0.3	1.1 ± 0.2	1.5 ± 0.4	2.0 ± 1.0	1.9 ± 0.5	2.0 ± 0.1	1.0 ± 0.2	1.0 ± 0.5	1.2 ± 0.5	1.1 ± 0.4	1.4 ± 0.2	1.2 ± 0.3
**Liver Function**												
AST	36.4 ± 17.7	36.5 ± 23.4	37.4 ± 15.4	36.9 ± 20.1	36.5 ± 15.6	38.4 ± 12.4	39.7 ± 11.5	38.4 ± 10.4	36.8 ± 12.7	39.4 ± 15.7	36.7 ± 15.4	39.4 ± 12.4
ALT	40.0 ± 8.8	39.4 ± 9.7	42.3 ± 10.4	42.6 ± 13.4	43.1 ± 13.7	39.8 ± 13.7	39.4 ± 10.4	42.7 ± 8.5	40.9 ± 7.7	42.1 ± 6.4	40.3 ± 7.5	40.2 ± 9.7
**Inflammation**												
Thrombomodulin (ng/mL)	56.3 ± 18.4	62.1 ± 20.1	123.5 ± 25.1 *	130.4 ± 21.0 *	49.7 ± 25.4	55.3 ± 19.7	110.3 ± 16.4 *	123.4 ± 19.7 *	52.1 ± 17.9	60.2 ± 19.4	50.1 ± 16.4	54.3 ± 12.7
CRP (mg/L)	3.0 ± 1.0	2.6 ± 1.2	9.0 ± 3.2	8.4 ± 2.1 *	2.9 ± 1.5	3.1 ± 1.4	5.1 ± 2.4	3.4 ± 1.0	3.2 ± 1.0	2.9 ± 1.4	4.1 ± 1.5	3.4 ± 1.0

BUN, blood urea nitrogen; AST, aspartate aminotransferase; ALT, alanine transaminase; CRP, C-reactive protein Values are mean ± SD. * *p* < 0.05 compared with pre-surgery at the same group.

**Table 5:** Perioperative characteristics (*n* = 347) in elective CABG patients.

Operation/Genotype	CABG surgery with Conventional CPB		CABG Surgery with Beating-Heart CPB		CABG Surgery with Off-Pump Technique	
	CC	CT/TT	CC	CT/TT	CC	CT/TT
pRBC transfusion (unit)	5.0 ± 2.7	5.2 ± 3.1	4.0 ± 2.6	4.1 ± 2.2	2.2 ± 1.2	2.5 ± 1.0
Drainage loss (mL)	986.1 ± 123.7	980.1 ± 104.7	382.6 ± 101.6 *	399.6 ± 121.8 #	330.7 ± 121.3 *	288.7 ± 91.9 #
ICU stay (*n*, %, average days)	4.0	5.0§	2.5 *	2.4 #	2.1 *	2.1 #
ICU fever (*n*, %, average days)	46, 39.7%, 3.2	49, 60.0% §, 4.9 §	14, 8.2% *, 2.0 *	3, 10.7% #, 2.0 #	5, 11.3% *, 2.1 *	4, 11.1% #, 2.1 #
Mortality after surgery for 48 h	0	0	0	0	0	0

pRBC, packed red blood cells; ICU fever: ear temperature ≥37.5 °C. Values are mean ± SD; * *p* < 0.05 compared with the CC genotype in CABG surgery with conventional CPB group; # *p* < 0.05 compared with the CT/TT genotype in CABG surgery with conventional CPB group; § *p* < 0.05 compared with pre-surgery in CABG surgery with conventional CPB group.

## Abbreviations

PCI	Percutaneous coronary intervention
CABG	coronary artery bridge graft
CPB	cardiopulmonary bypass
beating-heart CPB	on-pump beating-heart CPB
TM	Thrombomodulin
IABP	intra-aortic balloon pump
CK-MB	creatine kinase
BUN	blood urea nitrogen
AST	aspartate aminotransferase
ALT	alanine transaminase
CRP	C-reactive protein
pRBCs	packed red blood cells
VCAM-1	vascular cell adhesion molecule-1
